# Tris(4-hydroxy­pyridinium) hydrogen sulfate–sulfate monohydrate

**DOI:** 10.1107/S1600536809048545

**Published:** 2009-11-21

**Authors:** Ying-Ming Xu, Shan Gao, Seik Weng Ng

**Affiliations:** a College of Chemistry and Materials Science, Heilongjiang University, Harbin 150080, People’s Republic of China; b Department of Chemistry, University of Malaya, 50603 Kuala Lumpur, Malaysia

## Abstract

In the crystal structure of the title salt, 3C_5_H_6_NO^+^·HSO_4_^−^·SO_4_^2−^·H_2_O, the hydrogen sulfate ion is linked to the sulfate ion by an O—H⋯O hydrogen bond. The hydrogen sulfate–sulfate anion is a hydrogen-bond acceptor for the three independent cations and the uncoordinated water mol­ecule, the hydrogen-bonding inter­actions giving rise to a three-dimensional hydrogen-bonded network. In the hydrogen sulfate–sulfate species, one of the sulfate groups is disordered in respect of its O atoms in a 2:1 ratio.

## Related literature

For the crystal structure of bis­(4-hydroxy­pyridinium) sulfate monohydrate, see: Xu *et al.* (2009 ▶).
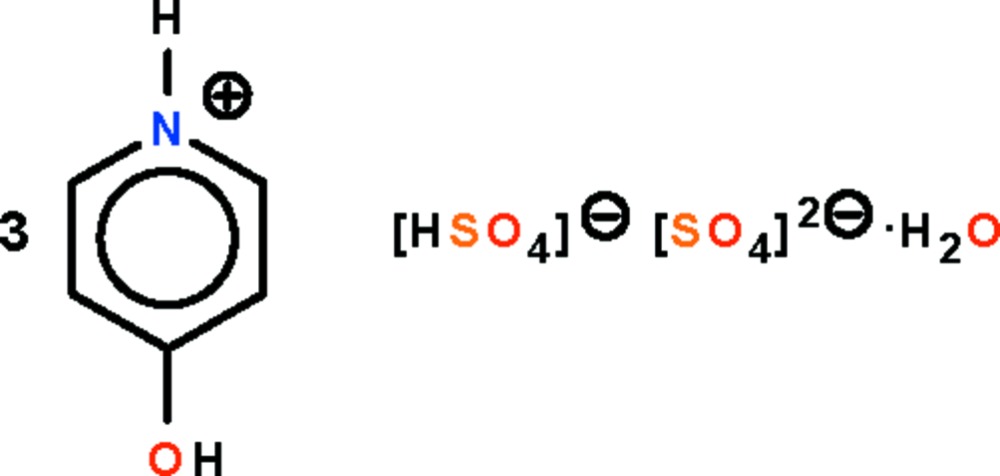


## Experimental

### 

#### Crystal data

3C_5_H_6_NO^+^·HSO_4_^−^·SO_4_^2−^·H_2_O
*M* *_r_* = 499.47
Orthorhombic, 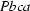

*a* = 10.5622 (3) Å
*b* = 19.6760 (7) Å
*c* = 20.2980 (7) Å
*V* = 4218.4 (2) Å^3^
*Z* = 8
Mo *K*α radiation
μ = 0.32 mm^−1^
*T* = 293 K
0.23 × 0.17 × 0.14 mm

#### Data collection

Rigaku R-AXIS RAPID IP diffractometer
Absorption correction: multi-scan (*ABSCOR*; Higashi, 1995 ▶) *T*_min_ = 0.930, *T*_max_ = 0.956
38906 measured reflections
4816 independent reflections
3031 reflections with *I* > 2σ(*I*)
*R*_int_ = 0.064

#### Refinement

*R*[*F* ^2^ > 2σ(*F* ^2^)] = 0.045
*wR*(*F* ^2^) = 0.133
*S* = 1.08
4816 reflections
359 parameters
151 restraints
H atoms treated by a mixture of independent and constrained refinement
Δρ_max_ = 0.36 e Å^−3^
Δρ_min_ = −0.31 e Å^−3^

### 

Data collection: *RAPID-AUTO* (Rigaku, 1998 ▶); cell refinement: *RAPID-AUTO*; data reduction: *CrystalClear* (Rigaku/MSC, 2002 ▶); program(s) used to solve structure: *SHELXS97* (Sheldrick, 2008 ▶); program(s) used to refine structure: *SHELXL97* (Sheldrick, 2008 ▶); molecular graphics: *X-SEED* (Barbour, 2001 ▶); software used to prepare material for publication: *publCIF* (Westrip, 2009 ▶).

## Supplementary Material

Crystal structure: contains datablocks global, I. DOI: 10.1107/S1600536809048545/xu2676sup1.cif

Structure factors: contains datablocks I. DOI: 10.1107/S1600536809048545/xu2676Isup2.hkl

Additional supplementary materials:  crystallographic information; 3D view; checkCIF report

## Figures and Tables

**Table 1:** Hydrogen-bond geometry (Å, °)

*D*—H⋯*A*	*D*—H	H⋯*A*	*D*⋯*A*	*D*—H⋯*A*
O2—H2o⋯O6	0.85	1.68	2.473 (3)	154
O6—H6o⋯O2	0.85	1.76	2.473 (3)	139
O9—H9o⋯O4^i^	0.86 (1)	1.76 (1)	2.612 (6)	174 (4)
O9—H9o⋯O4′^i^	0.86 (1)	1.72 (2)	2.569 (9)	172 (4)
O10—H10o⋯O1w^ii^	0.86 (1)	1.70 (1)	2.554 (3)	172 (3)
O11—H11o⋯O8^ii^	0.86 (1)	1.73 (1)	2.591 (3)	173 (4)
O1w—H1w⋯O7	0.85 (1)	1.91 (1)	2.754 (3)	173 (4)
O1w—H2w⋯O3^iii^	0.84 (1)	1.93 (2)	2.749 (4)	162 (4)
N1—H1n⋯O1	0.85 (1)	1.99 (2)	2.798 (3)	159 (3)
N1—H1n⋯O1′	0.85 (1)	2.23 (3)	2.907 (6)	138 (3)
N2—H2n⋯O5	0.85 (1)	1.96 (1)	2.795 (3)	169 (3)
N3—H3n⋯O7	0.85 (1)	1.94 (1)	2.768 (3)	167 (3)

Symmetry codes: (i) 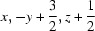; (ii) 
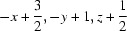
; (iii) 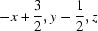.

**Fig. 1.**
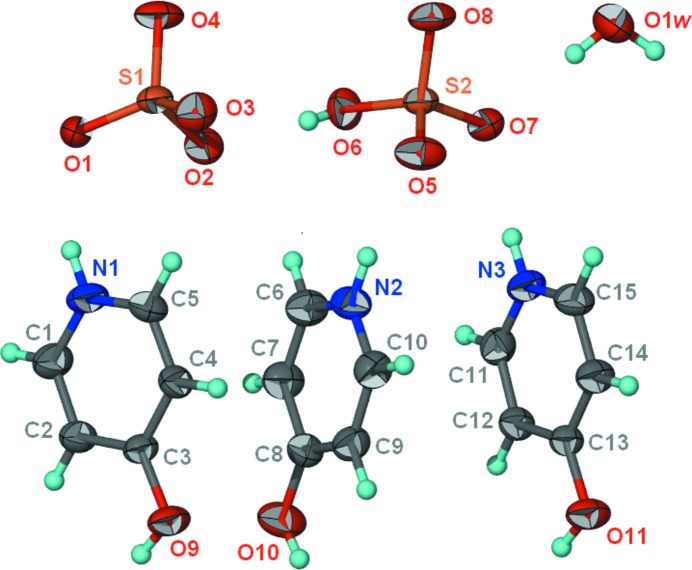
Thermal ellipsoid plot (Barbour, 2001) of 3[C5H6NO] [HSO4] [SO4].H2O at the 50% probability level; hydrogen atoms are drawn as spheres of arbitrary radius. The disorder in the sulfate is not shown.

## supplementary crystallographic information

### Experimental

Calcium chloride dihydrate (0.29 g, 2 mmol) and 4-hydroxypyridine-3-sulfonic acid (0.35 g, 2 mmol) were dissolved in hot water. The pH value was adjusted to 6 with 0.1 *M* sodium hydroxide. The solution was allowed to evaporate slowly at room temperature; colorless prismatic crystals were isolated from the clear solution after a few days.

### Refinement

One of the two independent sulfate ions is disordered over two positions. For the disorder ion, all sulfur–oxygen distances were restrained to within 0.01 Å of each other, as were the oxygen^···^oxygen distances. The aniosotropic temperature factors of the disordered oxygen atoms were restrained to be nearly isotropic. As the disordered refined to a 2:1 ratio, the ratio was then fixed as exactly 2:1.

Carbon-bound H-atoms were placed in calculated positions (C–H 0.93 Å) and were included in the refinement in the riding model approximation, with *U*(H) set to 1.2*U*(C). The amino and water H-atoms were located in a difference Fourier map, and were refined with a distance restraint of N–H = O–H = 0.85±0.01 Å; their temperature factors were refined. Additionally, for the water molecule, an H···H = 1.39 Å restrained was applied.

In the latter stages of the refinement, a hydrogen atom was located midway between one oxygen atom of the major-component sulfate ion and one oxygen atom of the ordered sulfate ion at a distance of 1.25 Å. This atom was then regarded as being 33% bonded to the first oxygen atoms and 67% bonded to the second oxygen atom. Although the two components could be refined, they were instead constrained to ride instead (O–H 0.85 Å).

### Crystal data

**Table:**

3C_5_H_6_NO^+^·HSO_4_^−^·SO_4_^2^^−^·H_2_O	*F*(000) = 2080
*M**_r_* = 499.47	*D*_x_ = 1.573 Mg m^−^^3^
Orthorhombic, *P**b**c**a*	Mo *K*α radiation, λ = 0.71073 Å
Hall symbol: -P 2ac 2ab	Cell parameters from 21088 reflections
*a* = 10.5622 (3) Å	θ = 3.0–27.5°
*b* = 19.6760 (7) Å	µ = 0.32 mm^−^^1^
*c* = 20.2980 (7) Å	*T* = 293 K
*V* = 4218.4 (2) Å^3^	Prism, colorless
*Z* = 8	0.23 × 0.17 × 0.14 mm

### Data collection

**Table:**

Rigaku R-AXIS RAPID IP diffractometer	4816 independent reflections
Radiation source: fine-focus sealed tube	3031 reflections with *I* > 2σ(*I*)
graphite	*R*_int_ = 0.064
ω scan	θ_max_ = 27.5°, θ_min_ = 3.0°
Absorption correction: multi-scan (*ABSCOR*; Higashi, 1995)	*h* = −13→13
*T*_min_ = 0.930, *T*_max_ = 0.956	*k* = −25→25
38906 measured reflections	*l* = −26→25

### Refinement

**Table:**

Refinement on *F*^2^	Primary atom site location: structure-invariant direct methods
Least-squares matrix: full	Secondary atom site location: difference Fourier map
*R*[*F*^2^ > 2σ(*F*^2^)] = 0.045	Hydrogen site location: inferred from neighbouring sites
*wR*(*F*^2^) = 0.133	H atoms treated by a mixture of independent and constrained refinement
*S* = 1.08	*w* = 1/[σ^2^(*F*_o_^2^) + (0.0687*P*)^2^] where *P* = (*F*_o_^2^ + 2*F*_c_^2^)/3
4816 reflections	(Δ/σ)_max_ = 0.001
359 parameters	Δρ_max_ = 0.36 e Å^−^^3^
151 restraints	Δρ_min_ = −0.31 e Å^−^^3^

### Fractional atomic coordinates and isotropic or equivalent isotropic displacement parameters (Å^2^)

**Table:**

	*x*	*y*	*z*	*U*_iso_*/*U*_eq_	Occ. (<1)
S1	0.47437 (6)	0.72762 (4)	0.42526 (3)	0.0411 (2)	
S2	0.70661 (6)	0.53636 (3)	0.42933 (3)	0.03703 (19)	
O1	0.3908 (3)	0.77692 (16)	0.45160 (13)	0.0526 (8)	0.67
O2	0.4603 (3)	0.66376 (14)	0.46657 (13)	0.0534 (7)	0.67
H2O	0.4899	0.6300	0.4455	0.080*	0.33
O3	0.6093 (2)	0.74804 (17)	0.43084 (14)	0.0526 (7)	0.67
O4	0.4463 (6)	0.7087 (3)	0.35765 (17)	0.057 (2)	0.67
O1'	0.4860 (6)	0.7986 (2)	0.4441 (3)	0.0619 (17)	0.33
O2'	0.3670 (5)	0.6974 (3)	0.4642 (3)	0.0687 (18)	0.33
O3'	0.5908 (4)	0.6914 (3)	0.4441 (3)	0.0594 (16)	0.33
O4'	0.4480 (10)	0.7201 (6)	0.3559 (3)	0.050 (4)	0.33
O5	0.79101 (16)	0.57990 (11)	0.46790 (8)	0.0564 (5)	
O6	0.57405 (16)	0.55782 (10)	0.43845 (10)	0.0520 (5)	
H6O	0.5716	0.6002	0.4464	0.078*	0.67
O7	0.71622 (18)	0.46548 (9)	0.45195 (9)	0.0516 (5)	
O8	0.73894 (17)	0.53979 (11)	0.35927 (8)	0.0557 (5)	
O9	0.61528 (17)	0.78290 (11)	0.76401 (9)	0.0525 (5)	
O10	0.5361 (2)	0.61193 (12)	0.77445 (10)	0.0622 (6)	
O11	0.92913 (18)	0.45490 (11)	0.76645 (9)	0.0516 (5)	
O1W	0.8138 (3)	0.36629 (14)	0.37081 (11)	0.0745 (7)	
N1	0.4461 (2)	0.79165 (14)	0.58566 (11)	0.0523 (6)	
N2	0.6911 (2)	0.60986 (12)	0.59204 (11)	0.0477 (6)	
N3	0.7757 (2)	0.44865 (13)	0.58375 (11)	0.0486 (6)	
C1	0.3737 (3)	0.79553 (16)	0.63918 (13)	0.0526 (7)	
H1	0.2866	0.8006	0.6346	0.063*	
C2	0.4252 (2)	0.79225 (14)	0.70003 (12)	0.0442 (6)	
H2	0.3740	0.7943	0.7372	0.053*	
C3	0.5562 (2)	0.78579 (13)	0.70632 (11)	0.0377 (6)	
C4	0.6297 (2)	0.78230 (13)	0.64949 (12)	0.0406 (6)	
H4	0.7173	0.7782	0.6524	0.049*	
C5	0.5724 (3)	0.78489 (14)	0.58982 (12)	0.0466 (7)	
H5	0.6207	0.7820	0.5516	0.056*	
C6	0.5672 (3)	0.59812 (15)	0.60013 (14)	0.0514 (7)	
H6	0.5166	0.5892	0.5636	0.062*	
C7	0.5147 (2)	0.59914 (15)	0.66110 (13)	0.0512 (7)	
H7	0.4285	0.5912	0.6663	0.061*	
C8	0.5901 (2)	0.61199 (14)	0.71564 (12)	0.0434 (6)	
C9	0.7191 (2)	0.62398 (14)	0.70587 (13)	0.0449 (6)	
H9	0.7718	0.6329	0.7415	0.054*	
C10	0.7664 (3)	0.62247 (14)	0.64387 (13)	0.0461 (7)	
H10	0.8522	0.6303	0.6371	0.055*	
C11	0.6988 (2)	0.44270 (14)	0.63582 (13)	0.0466 (7)	
H11	0.6123	0.4373	0.6293	0.056*	
C12	0.7455 (2)	0.44448 (13)	0.69802 (12)	0.0402 (6)	
H12	0.6915	0.4401	0.7339	0.048*	
C13	0.8755 (2)	0.45294 (13)	0.70767 (11)	0.0367 (6)	
C14	0.9534 (2)	0.45910 (14)	0.65243 (12)	0.0447 (6)	
H14	1.0403	0.4647	0.6574	0.054*	
C15	0.9007 (3)	0.45679 (15)	0.59115 (13)	0.0489 (7)	
H15	0.9521	0.4609	0.5542	0.059*	
H1N	0.415 (3)	0.7950 (17)	0.5474 (8)	0.082 (11)*	
H2N	0.721 (3)	0.6063 (15)	0.5534 (7)	0.062 (9)*	
H3N	0.747 (3)	0.4507 (18)	0.5449 (8)	0.088 (12)*	
H1W	0.789 (3)	0.3988 (12)	0.3951 (14)	0.093 (13)*	
H2W	0.848 (4)	0.3357 (14)	0.3939 (15)	0.115 (17)*	
H9O	0.556 (2)	0.7852 (18)	0.7929 (14)	0.089 (13)*	
H10O	0.592 (2)	0.6200 (17)	0.8044 (12)	0.076 (12)*	
H11O	0.869 (2)	0.4553 (18)	0.7952 (13)	0.087 (12)*	

### Atomic displacement parameters (Å^2^)

**Table:**

	*U*^11^	*U*^22^	*U*^33^	*U*^12^	*U*^13^	*U*^23^
S1	0.0440 (4)	0.0531 (4)	0.0263 (3)	0.0036 (3)	−0.0035 (3)	−0.0001 (3)
S2	0.0342 (3)	0.0518 (4)	0.0250 (3)	−0.0001 (3)	−0.0003 (2)	0.0013 (3)
O1	0.0608 (19)	0.063 (2)	0.0335 (14)	0.0284 (16)	−0.0029 (14)	−0.0034 (14)
O2	0.0640 (19)	0.0522 (19)	0.0440 (16)	0.0061 (16)	0.0156 (14)	0.0115 (13)
O3	0.0423 (15)	0.063 (2)	0.0523 (17)	−0.0059 (15)	−0.0035 (13)	0.0008 (15)
O4	0.059 (4)	0.085 (4)	0.028 (3)	−0.009 (3)	−0.003 (3)	−0.006 (2)
O1'	0.086 (5)	0.048 (4)	0.052 (3)	0.000 (3)	−0.018 (3)	−0.006 (3)
O2'	0.054 (4)	0.107 (5)	0.045 (3)	−0.016 (4)	0.010 (3)	0.005 (3)
O3'	0.043 (3)	0.067 (4)	0.068 (4)	0.016 (3)	−0.017 (3)	−0.005 (3)
O4'	0.038 (6)	0.088 (7)	0.023 (6)	0.007 (5)	−0.001 (5)	−0.007 (5)
O5	0.0520 (11)	0.0785 (15)	0.0388 (10)	−0.0227 (10)	−0.0029 (8)	−0.0068 (10)
O6	0.0376 (10)	0.0588 (13)	0.0597 (12)	0.0082 (9)	0.0023 (9)	−0.0031 (10)
O7	0.0676 (12)	0.0504 (12)	0.0368 (10)	0.0132 (9)	−0.0048 (9)	0.0025 (8)
O8	0.0460 (10)	0.0943 (16)	0.0268 (9)	−0.0045 (10)	0.0017 (8)	0.0067 (9)
O9	0.0397 (10)	0.0872 (16)	0.0306 (10)	0.0037 (10)	−0.0050 (8)	0.0019 (9)
O10	0.0547 (13)	0.0897 (17)	0.0422 (11)	−0.0062 (12)	0.0121 (10)	−0.0051 (11)
O11	0.0415 (10)	0.0806 (15)	0.0326 (10)	−0.0052 (10)	−0.0041 (8)	−0.0032 (9)
O1W	0.1018 (19)	0.0718 (18)	0.0498 (13)	0.0291 (15)	0.0102 (13)	−0.0019 (12)
N1	0.0538 (14)	0.0726 (18)	0.0306 (12)	0.0034 (13)	−0.0085 (10)	0.0002 (11)
N2	0.0551 (15)	0.0519 (15)	0.0361 (12)	−0.0027 (11)	0.0071 (11)	0.0024 (11)
N3	0.0600 (15)	0.0511 (15)	0.0346 (12)	0.0046 (12)	−0.0121 (11)	0.0001 (10)
C1	0.0375 (14)	0.076 (2)	0.0438 (15)	0.0026 (14)	−0.0051 (12)	0.0008 (14)
C2	0.0378 (14)	0.0608 (18)	0.0339 (13)	0.0014 (12)	0.0016 (11)	−0.0008 (12)
C3	0.0383 (13)	0.0455 (16)	0.0292 (12)	0.0000 (11)	−0.0035 (10)	0.0012 (10)
C4	0.0366 (13)	0.0484 (16)	0.0369 (13)	0.0025 (11)	0.0035 (11)	−0.0019 (11)
C5	0.0537 (16)	0.0541 (18)	0.0320 (13)	0.0028 (13)	0.0066 (12)	−0.0020 (11)
C6	0.0482 (16)	0.0606 (19)	0.0453 (15)	−0.0073 (14)	−0.0031 (13)	−0.0012 (14)
C7	0.0413 (14)	0.064 (2)	0.0479 (16)	−0.0049 (14)	0.0012 (12)	−0.0013 (14)
C8	0.0452 (15)	0.0466 (16)	0.0384 (14)	0.0018 (12)	0.0048 (11)	0.0001 (12)
C9	0.0407 (14)	0.0531 (18)	0.0409 (14)	−0.0012 (12)	−0.0039 (11)	0.0020 (12)
C10	0.0414 (14)	0.0493 (17)	0.0476 (16)	−0.0043 (12)	0.0027 (12)	0.0052 (12)
C11	0.0386 (14)	0.0513 (18)	0.0498 (16)	0.0022 (12)	−0.0077 (12)	−0.0040 (13)
C12	0.0356 (13)	0.0473 (16)	0.0378 (13)	−0.0021 (11)	0.0023 (11)	−0.0016 (12)
C13	0.0367 (13)	0.0413 (15)	0.0321 (12)	−0.0008 (11)	−0.0009 (10)	−0.0014 (10)
C14	0.0375 (13)	0.0581 (18)	0.0386 (14)	−0.0031 (12)	0.0006 (11)	0.0020 (12)
C15	0.0572 (18)	0.0556 (18)	0.0340 (13)	−0.0003 (14)	0.0066 (12)	0.0031 (12)

### Geometric parameters (Å, °)

**Table:**

S1—O1	1.416 (2)	N3—C11	1.338 (3)
S1—O4'	1.444 (5)	N3—C15	1.339 (4)
S1—O4	1.452 (3)	N3—H3N	0.847 (11)
S1—O1'	1.453 (4)	C1—C2	1.351 (3)
S1—O3'	1.473 (4)	C1—H1	0.9300
S1—O3	1.485 (2)	C2—C3	1.396 (3)
S1—O2'	1.505 (4)	C2—H2	0.9300
S1—O2	1.518 (3)	C3—C4	1.392 (3)
S2—O5	1.4633 (18)	C4—C5	1.355 (3)
S2—O8	1.4641 (17)	C4—H4	0.9300
S2—O7	1.4717 (19)	C5—H5	0.9300
S2—O6	1.4740 (18)	C6—C7	1.356 (4)
O2—H2O	0.8501	C6—H6	0.9300
O6—H6O	0.8501	C7—C8	1.386 (3)
O9—C3	1.328 (3)	C7—H7	0.9300
O9—H9O	0.860 (11)	C8—C9	1.397 (3)
O10—C8	1.323 (3)	C9—C10	1.355 (3)
O10—H10O	0.862 (11)	C9—H9	0.9300
O11—C13	1.321 (3)	C10—H10	0.9300
O11—H11O	0.863 (11)	C11—C12	1.356 (3)
O1W—H1W	0.847 (11)	C11—H11	0.9300
O1W—H2W	0.844 (11)	C12—C13	1.397 (3)
N1—C1	1.330 (3)	C12—H12	0.9300
N1—C5	1.343 (4)	C13—C14	1.396 (3)
N1—H1N	0.846 (11)	C14—C15	1.363 (3)
N2—C6	1.339 (3)	C14—H14	0.9300
N2—C10	1.342 (3)	C15—H15	0.9300
N2—H2N	0.848 (11)		
			
O4'—S1—O1'	111.8 (5)	O9—C3—C4	117.8 (2)
O4—S1—O1'	120.8 (4)	O9—C3—C2	123.4 (2)
O4'—S1—O3'	111.4 (4)	C4—C3—C2	118.8 (2)
O1'—S1—O3'	109.1 (3)	C5—C4—C3	119.3 (2)
O1—S1—O3	112.58 (19)	C5—C4—H4	120.3
O4—S1—O3	109.7 (3)	C3—C4—H4	120.3
O4'—S1—O2'	109.1 (4)	N1—C5—C4	120.2 (2)
O1'—S1—O2'	107.8 (3)	N1—C5—H5	119.9
O3'—S1—O2'	107.5 (3)	C4—C5—H5	119.9
O1—S1—O2	107.29 (17)	N2—C6—C7	120.6 (3)
O4—S1—O2	106.8 (3)	N2—C6—H6	119.7
O3—S1—O2	106.03 (17)	C7—C6—H6	119.7
O5—S2—O8	110.52 (11)	C6—C7—C8	119.8 (2)
O5—S2—O7	110.23 (12)	C6—C7—H7	120.1
O8—S2—O7	109.31 (12)	C8—C7—H7	120.1
O5—S2—O6	110.11 (12)	O10—C8—C7	118.2 (2)
O8—S2—O6	109.29 (11)	O10—C8—C9	123.3 (2)
O7—S2—O6	107.32 (11)	C7—C8—C9	118.5 (2)
S1—O2—H2O	109.5	C10—C9—C8	119.2 (2)
S2—O6—H6O	109.5	C10—C9—H9	120.4
C3—O9—H9O	105 (2)	C8—C9—H9	120.4
C8—O10—H10O	110 (2)	N2—C10—C9	120.9 (3)
C13—O11—H11O	107 (2)	N2—C10—H10	119.5
H1w—O1w—H2W	110.3 (18)	C9—C10—H10	119.5
C1—N1—C5	121.7 (2)	N3—C11—C12	120.9 (2)
C1—N1—H1N	122 (2)	N3—C11—H11	119.6
C5—N1—H1N	117 (2)	C12—C11—H11	119.6
C6—N2—C10	121.0 (2)	C11—C12—C13	119.4 (2)
C6—N2—H2N	118 (2)	C11—C12—H12	120.3
C10—N2—H2N	121 (2)	C13—C12—H12	120.3
C11—N3—C15	121.3 (2)	O11—C13—C14	118.1 (2)
C11—N3—H3N	121 (2)	O11—C13—C12	123.5 (2)
C15—N3—H3N	117 (2)	C14—C13—C12	118.5 (2)
N1—C1—C2	120.9 (2)	C15—C14—C13	119.3 (2)
N1—C1—H1	119.6	C15—C14—H14	120.3
C2—C1—H1	119.6	C13—C14—H14	120.3
C1—C2—C3	119.1 (2)	N3—C15—C14	120.6 (2)
C1—C2—H2	120.4	N3—C15—H15	119.7
C3—C2—H2	120.4	C14—C15—H15	119.7
			
C5—N1—C1—C2	−0.5 (5)	O10—C8—C9—C10	179.4 (3)
N1—C1—C2—C3	1.0 (4)	C7—C8—C9—C10	−0.2 (4)
C1—C2—C3—O9	179.3 (3)	C6—N2—C10—C9	−0.3 (4)
C1—C2—C3—C4	−0.6 (4)	C8—C9—C10—N2	0.2 (4)
O9—C3—C4—C5	179.8 (2)	C15—N3—C11—C12	−0.3 (4)
C2—C3—C4—C5	−0.3 (4)	N3—C11—C12—C13	0.3 (4)
C1—N1—C5—C4	−0.5 (4)	C11—C12—C13—O11	−179.6 (3)
C3—C4—C5—N1	0.9 (4)	C11—C12—C13—C14	−0.2 (4)
C10—N2—C6—C7	0.4 (4)	O11—C13—C14—C15	179.6 (2)
N2—C6—C7—C8	−0.4 (5)	C12—C13—C14—C15	0.1 (4)
C6—C7—C8—O10	−179.3 (3)	C11—N3—C15—C14	0.2 (4)
C6—C7—C8—C9	0.4 (4)	C13—C14—C15—N3	−0.1 (4)

### Hydrogen-bond geometry (Å, °)

**Table:**

*D*—H···*A*	*D*—H	H···*A*	*D*···*A*	*D*—H···*A*
O2—H2o···O6	0.85	1.68	2.473 (3)	154
O6—H6o···O2	0.85	1.76	2.473 (3)	139
O9—H9o···O4^i^	0.86 (1)	1.76 (1)	2.612 (6)	174 (4)
O9—H9o···O4'^i^	0.86 (1)	1.72 (2)	2.569 (9)	172 (4)
O10—H10o···O1w^ii^	0.86 (1)	1.70 (1)	2.554 (3)	172 (3)
O11—H11o···O8^ii^	0.86 (1)	1.73 (1)	2.591 (3)	173 (4)
O1w—H1w···O7	0.85 (1)	1.91 (1)	2.754 (3)	173 (4)
O1w—H2w···O3^iii^	0.84 (1)	1.93 (2)	2.749 (4)	162 (4)
N1—H1n···O1	0.85 (1)	1.99 (2)	2.798 (3)	159 (3)
N1—H1n···O1'	0.85 (1)	2.23 (3)	2.907 (6)	138 (3)
N2—H2n···O5	0.85 (1)	1.96 (1)	2.795 (3)	169 (3)
N3—H3n···O7	0.85 (1)	1.94 (1)	2.768 (3)	167 (3)

Symmetry codes: (i) *x*, −*y*+3/2, *z*+1/2; (ii) −*x*+3/2, −*y*+1, *z*+1/2; (iii) −*x*+3/2, *y*−1/2, *z*.
